# Cities and regions in Britain through hierarchical percolation

**DOI:** 10.1098/rsos.150691

**Published:** 2016-04-06

**Authors:** Elsa Arcaute, Carlos Molinero, Erez Hatna, Roberto Murcio, Camilo Vargas-Ruiz, A. Paolo Masucci, Michael Batty

**Affiliations:** 1 Centre for Advanced Spatial Analysis (CASA), University College London, London, UK; 2 Consumer Research Data Centre, Geography, University College London, London, UK; 3 Center for Advanced Modeling, The Johns Hopkins University, Baltimore, MD, USA

**Keywords:** percolation theory, urban hierarchies, city boundaries, fractal dimension, street networks

## Abstract

Urban systems present hierarchical structures at many different scales. These are observed as administrative regional delimitations which are the outcome of complex geographical, political and historical processes which leave almost indelible footprints on infrastructure such as the street network. In this work, we uncover a set of hierarchies in Britain at different scales using percolation theory on the street network and on its intersections which are the primary points of interaction and urban agglomeration. At the larger scales, the observed hierarchical structures can be interpreted as regional fractures of Britain, observed in various forms, from natural boundaries, such as National Parks, to regional divisions based on social class and wealth such as the well-known North–South divide. At smaller scales, cities are generated through recursive percolations on each of the emerging regional clusters. We examine the evolution of the morphology of the system as a whole, by measuring the fractal dimension of the clusters at each distance threshold in the percolation. We observe that this reaches a maximum plateau at a specific distance. The clusters defined at this distance threshold are in excellent correspondence with the boundaries of cities recovered from satellite images, and from previous methods using population density.

## 1. Introduction

Countries are the historical outcome of the unification and the fragmentation of regions and communities. Although many of these processes are the result of an imposed organization devised through administrative boundaries, some others hold communities together through strong ideological ties at the regional level, creating a strong sense of belonging. These processes are intricate cultural, political and socio-historical pathways, that have left footprints in the way communities emerge, organize, trade and change spatially. These footprints are contained in the street patterns and network, which are the main proxies for communication and exchange between settlements. It is hence not surprising that these also encompass the socio-economic history of the region. Despite the constant change and renewal of streets, we show in this work, that these footprints can still be identified and recovered.

We focus on Britain, whose road network has been evolving for over 2000 years. Its origins can be traced back to the Iron Age with the Celts, but it was during the Roman occupation that a rapid expansion of the roads took place, and a network was established. In the last 200 years, this has been subjected to ongoing urban growth, and to adaptations for new extensions and modes of transport. Britain presents a rich regional structure, whose tensions lead to a fractured landscape, driven by ideologies and socio-historical trajectories. One example is the surge for the need to call for a referendum on Scottish independence (2014), and the rise of the Scottish Nationalists in the recent elections of 2014–2015. Discerning the emergent fractions that are independent of administrative provisions, but highly tied to their trajectories and ideologies, is of considerable relevance for the understanding of the dynamical regional functioning of a country. In a different paper [1], we corroborate the high correlation between regional divisions and polarization represented through voting patterns.

In this work, we investigate whether the regional fractures of Britain can be observed through an underlying hierarchical structure which can be recovered from the road network. From the beginnings of locational analysis [2], there has been an understanding that regional organization can be perceived through movement, and that the expansion of infrastructural networks is tightly linked to regional development. In this sense, the hierarchical perspective can be understood from the economic performance of regions, leaving traces in the road network’s evolution. The quest to characterize and quantify the regionalization of the urban space in a hierarchical manner, dates back to the 1930s. Initial ideas focused on subdividing the space, considering on the one hand morphological characteristics, and on the other population distribution. These were proposed in various contexts, from regular lattices [3], to elaborated structures, such as the ones proposed by Christaller [4] in his Central-Place hierarchies. Later on, efforts were directed into the hierarchical aspect of distance between settlements [5,6], where a direct correlation between the size of a cluster and the distance to other clusters was identified [7]. This was extended in the 1960s, to a perspective on the functional characterization of clusters according to size. A hierarchy emerges with respect to the types of relationships that exist given the cluster size (whether the cluster is a village, a town or a city) [8–10]. These translate in contemporary urban studies to the study of scaling laws [11,12], where nevertheless, it is assumed that all types emerge, and the hierarchy is indirectly linked with the aggregated value of the urban measurement, determined by the size of the cluster. It seems, that there is a need for a careful re-evaluation of the characterization of urban indicators at various regimes of size. Coming back to the hierarchical approach to urban functionalities, this has paved the way for different perspectives on urban modelling [13–17]. Currently, network theory has been extremely successful and permeates the methodologies employed for many different urban models. This approach nevertheless, dates back to the 1960s, where graph theory was used to establish a hierarchical structure between regions, through the introduction of a measure of flow between places, from telephone calls to trade [18].

A recurrent aspect in the above-mentioned approaches which decode urban hierarchies is the connectivity of the system. This can be explored through percolation theory [19,20], which studies how a piece of information (or a disease, or a fire, etc.) spreads in space, reaching a critical point at which a giant cluster appears. In its most general form, the process is defined in an infinite lattice and for a random occupation probability. Relaxing these constraints, the analysis can be extended to finite systems, where the clusters are the outcome of some thresholding process. Some of these systems present a multiplicity of percolation transitions, revealing a hierarchical organization. This was observed for the brain [21], where the percolation process is considered in terms of the connectivity between voxels given by the different stimuli thresholds.

A crude analogy can be drawn between the structure of the brain and that of an urban system. Both consist of highly integrated modules which connect to each other at different scales, giving rise to a functional system. For the urban system, the modules correspond to its cities, and its different regional divisions are a manifestation of its inherent hierarchical structure [2,22]. We hence implement a similar methodology to [21] on the street intersections of Britain, in order to unveil its hierarchical organization. Note that the network has been stripped away, and the percolation process is hence applied to the street intersections, which correspond to the occupied sites in space, connected to each other through proximity only. Using the intersection points as a proxy for urbanization can be justified from archaeological times. In Anglo-Saxon Britain [23], the assembly places were defined at the main points of convergence, where the relevant interactions took place. In contemporary times, it has been argued that the road intersections are the essential facilitators for the necessary human dynamics that lead to a productive urban system [24]. In addition, there is also a technical bias that we purge ourselves from when removing the links of the network. This relates to the long-standing problems of digitization of the dataset: faulty topology of the network, missing streets, disconnected networks given by the inaccuracy of streets almost meeting, etc. In any case, for the skeptical reader, we also provide a similar methodology developed directly on the entire street network, which has been carefully prepared and checked in order to avoid many of the above-mentioned problems, and we show that the results are equally recovered.

In the following sections, we show that through a multiplicity of percolation transitions, the hierarchical structure of Britain emerges. These transitions indicate fractures of various sorts, from natural barriers, such as National Parks or lakes, to socio-economic polarization such as the North–South divide. The transition observed at the smallest scale defines the cores of the cities. It is well known that the morphological properties of cities and regions are notably different. These have been extensively analysed for street networks [25–29], nevertheless, the statistical properties previously found cannot be used to define the boundaries of a city, since there is no clear transition between urban and rural networks. Here, we show that through the analysis of the fractal dimension of the emergent clusters, a threshold can be identified at which cities are well defined. This specific morphological property observed over the whole system, gives a maximum over all the thresholds. At this maximum, the obtained clusters are in very good correspondence with other proxies for cities, such as satellite images of the urbanized space, and previous definitions of cities proposed by the authors [12].

## 2. Methodology and dataset

Percolation theory is classically approached in terms of the probability of a site being occupied in a lattice. It can also be thought of in terms of bond percolation, in which the sites are all occupied, and the probability corresponds to a bond to be open and to connect sites. In our analysis, the sites will correspond to the intersection points.

In the following section, we present two methodologies: (i) the percolation on the intersection points and (ii) the percolation on the street network. For both methods, we use the most complete database for street networks in Britain: the Ordnance Survey (OS) MasterMap [30]. For computational purposes, we reduce the size of the dataset by introducing the following simplifications: (i) we remove the points that do not convey any morphological information, such as nodes of degree two, which for example correspond to streets changing name; and (ii) we replace roundabouts by a single intersection point, which is primarily relevant for the methodology on networks. The original dataset contains more than 23 million points and the final processed dataset contains only around 3 million intersection points. In detail, the network covering the whole of the UK has *n*=3 390 758 nodes; *m*=3 973 186 links and the average node degree is 〈*k*〉=2.34.

### 2.1 Percolation on the street intersections

For this method, we take the dataset described above, and we remove all the street segments, leaving only the intersection points. We then apply a clustering algorithm that corresponds to a thresholding procedure parametrized by distance. This is simply defined as the Euclidean distance between points, whether they are connected or not. We observe different configurations of clusters appearing at different distances. This procedure can be interpreted in terms of bond percolation as follows: the probability of a bond to be open between sites, corresponds to the distance between the intersection points. In this sense, one can think of a fully connected network in which the distance between nodes gives the probability for the link to exist after a normalization procedure.

In practical terms, the algorithm is similar to the City Clustering Algorithm [31,32] based on population distribution in space, and the *natural cities* definition given also in terms of road intersections [33]. In [34], this algorithm is also employed to understand the emergence of regions through percolation theory, and in [35], in order to understand the spread of obesity in the USA. It is important to note that most of these algorithms have been constructed in an effort to define cities in a consistent way, and considerable research is still undergoing in this direction [36,37]. These algorithms differ from models of urban growth based on correlated percolation [38–40], and on correlations with urban sprawl [41].

In detail, our algorithm is defined in terms of a distance parameter that determines clusters of intersection points in which every point has a neighbour at a distance equal or smaller than the given threshold. The algorithm can be implemented on the continuous space, or for large datasets requiring computationally demanding calculations, on a grid covering the space of points. Refer to the electronic supplementary material, appendix A for more detail on the implementation of the algorithm.

### 2.2 Percolation on the network

In this case, we are considering the ‘real’ network, where intersection points are connected if and only if there is a street connecting them. The clustering procedure is very similar to the procedure described above, but in this case the distance is given by the actual extent of the street. An open bond hence corresponds in this case to an existing street according to the different distance thresholds. Once again, the links can be re-interpreted in terms of probabilities if the distances are normalized. Details on how to implement this can be found in the electronic supplementary material, appendix B.

## 3. Results

### 3.1 Urban hierarchies

**Figure 1.**
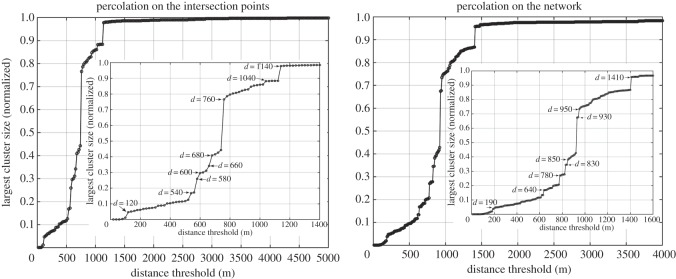
Evolution of the largest cluster size for the percolation on both systems. The size has been normalized by the total number of intersection points present in the dataset.

**Figure 2.**
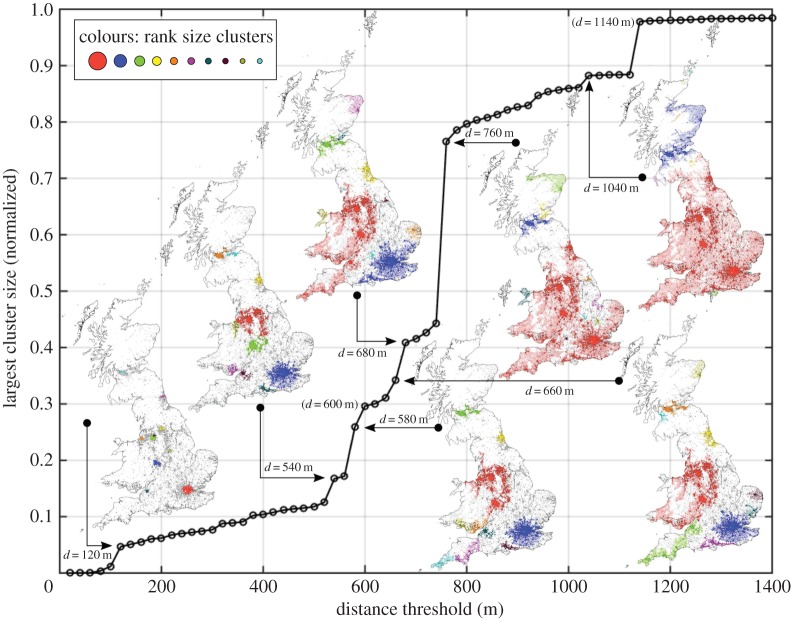
Evolution of the largest cluster size for the percolation on the intersection points.

We analyse the process in the traditional way, by looking at the size evolution of the largest cluster at the different distance thresholds [19]. A multiplicity of percolation transitions defining the fractures at different scales is observed. The same divisions can be found in both systems, see figure 1, although the critical distance threshold varies. As with any percolation process, the critical threshold that determines the transition pertains to the geometrical properties of the space. In the first system, the percolation occurs on a regular grid, while in the second system, the process takes place on the network itself. We will present the rest of the results for the first scenario, hence all the critical distances correspond to those observed for the points of intersection and are illustrated in figure 2. Details of the maps can be found in the electronic supplementary material, appendix C figure S1.

The first transition detected at *d*_*c*_=120 m denotes the merging of the north and south parts of London separated by the river Thames. The giant cluster can be identified with the core of London. Looking across the system, other large clusters correspond to the cores of important cities, such as Birmingham, Manchester, Liverpool, Bristol, etc. This first transition is therefore representative for a system of cities. Nevertheless, although the cores are recovered, the extent of the cities is much smaller than expected. In the next section, we will introduce a fractal analysis of the clusters at each distance threshold, and we will show that the system maximizes the value of the fractal dimension at the highest point of correlation with the urbanized space, defined through a classification of satellite images.

The giant cluster at the next transition, *d*_*c*_=540 m, encompasses the main post-industrial cities in the north: Liverpool, Manchester, Leeds and Sheffield. These are the main core cities conforming the region denominated as the ‘Northern Powerhouse’ (devolution proposal),^1^ with some others, such as Newcastle missing in the cluster. The Northern Powerhouse proposal aims at devolving power and at boosting economic growth in cities in the north, reducing the gap of wealth between these and the cities in the Southeast, mainly driven by London [45]. Such a disparity in wealth distribution dates back to Roman times, and saw an intensification after de-industrialization. Many claim that the reforms introduced by Margaret Thatcher’s Conservative governments during the 1980s made the North–South divide even more significant.

**Figure 3.**
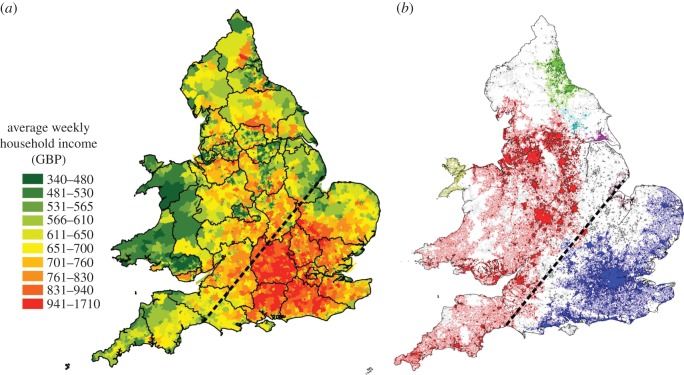
Maps of England and Wales: (*a*) thematic map of income with regional divisions given by NUTS2; (*b*) at percolation distance threshold *d*=740 m.

The next transition at *d*_*c*_=580 m, sees an expansion of that region, introducing Birmingham into the largest cluster. The following smaller transitions see the annexing of Wales into the cluster at *d*_*c*_=660 m, and at *d*_*c*_=680 m, that of Cornwall. At *d*=740 m before the next transition takes place, a very clear division between the Northwest and the Southeast can be observed. It is important to note at this point that such a division is not the outcome of a geographical accident of sorts, say the presence of natural barriers such as parks, mountains or rivers. In addition, it is not an artifice of taking only the intersection points, since the split is also present when the percolation is performed directly on the network. This split does not seem to be linked to the topography of the country, but to the division of wealth that we have been discussing throughout. We illustrate this in figure 3. The map on the right shows the 10 largest clusters in colour at *d*=740 m. The map on the left shows model-based income estimates for England and Wales at the level of Middle Layer Super Output Areas for 2007/2008. These are based on census data, and hence Scotland is excluded from this map, since it has a different census to England and Wales. The black boundaries correspond to European administrative regional divisions called NUTS2 [46]. The dotted lines indicate a clear agreement between the clusters obtained from the percolation process, and the division of wealth in the country.

At *d*_*c*_=760 m, England and Wales, with the exception of Snowdonia (region of mountains and a National Park in Wales), merge into one region, marking the most striking transition. This is followed by smaller transitions resulting from the merging of areas with natural barriers. At *d*=1120 m, Scotland can clearly be distinguished as a separate region from the rest of England and Wales. Note that the split of Scotland from the rest of the country is not caused by barriers of topographic nature either. This division is of a similar type to the one encountered earlier, it is the product of a historical cultural differentiation, that has been imprinted in the evolution of the infrastructure of the country. The last important transition in the system is observed at *d*_*c*_=1140 m, and it corresponds to the merging of Scotland with England and Wales.

It can be argued that these results are biased, given that the connectivity between points does not take into account whether these points can or cannot be connected through roads, because these were removed. To reassure the reader, we perform the percolation on the road network and we present the results in the electronic supplementary material, appendix C. The plots and maps, see figure S2, confirm the previous analysis, although at different critical distances. These are larger, as they correspond to the length of the roads individuals need to take to travel from one intersection point to another in the urban system. An important result of percolation theory, is that the value of the critical distances will vary from dataset to dataset, and from system to system. We hence do not expect to recover the same distances for the UK if a different dataset is used, nor the same distances for different countries.

**Figure 4.**
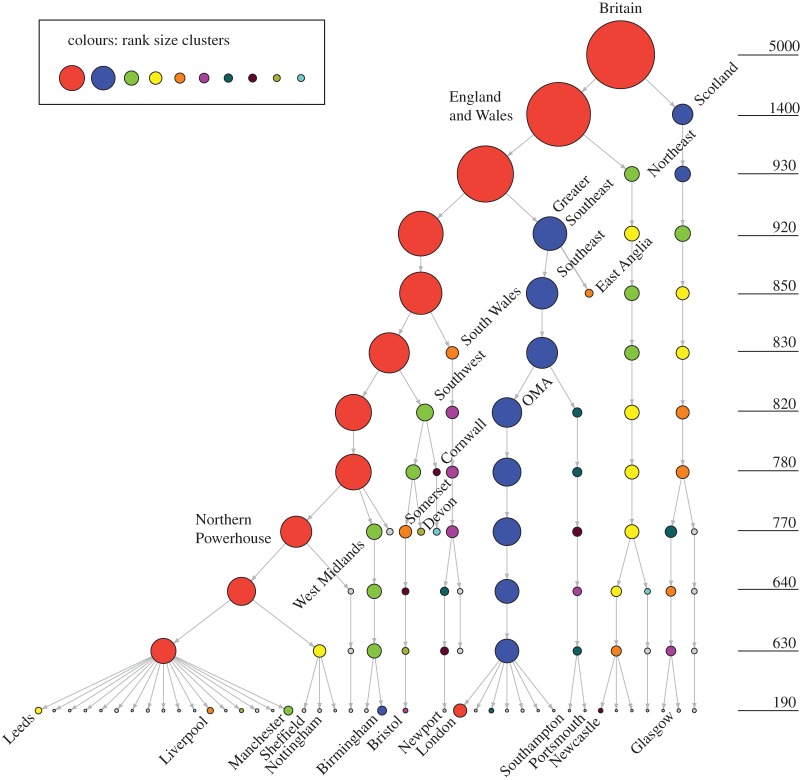
Dendogram of the evolution of some of the largest clusters through the percolation process. The size is measured according to the number of intersection points.

The results can be summarized in a crude way, by looking at the evolution of the relevant largest clusters through a dendogram (figure 4). The hierarchical structure becomes very tangible, and the size of the nodes are scaled to represent the size of the real clusters.

### 3.2 Fractal properties

Urbanized spaces have specific morphological properties that cannot be found in non-urbanized areas, and many of these are manifested in the road network. We investigate in this section, whether the clusters defined at each distance threshold can characterize the urban system in a particular morphological manner. We choose the fractal dimension as the property to analyse, given that this has been extensively researched for the morphological description of cities [47–49]. In [50] for example, a fractal analysis was undertaken to compare built-up areas from the Corine dataset [51] at different built-up densities for 20 European cities.

This section contains two subsections for the analysis of each of the systems, since the fractal dimension cannot be computed in the same way for point patterns and for a network.

#### 3.2.1 Clusters of intersection points

Until recently, the characterization of the fractal structure of a system consisted of a single fractal dimension. For cities, this was traditionally obtained through a box counting algorithm. Nevertheless, this measure is extremely sensitive to the dataset and the implementation. In addition, it is well recognized now, that cities are actually *multifractals* [52,53]. These are objects that present different fractal properties at different scales and regions [54], and hence cannot be fully characterized by a single fractal dimension, but need a spectrum of fractal dimensions [55–57].

**Figure 5.**
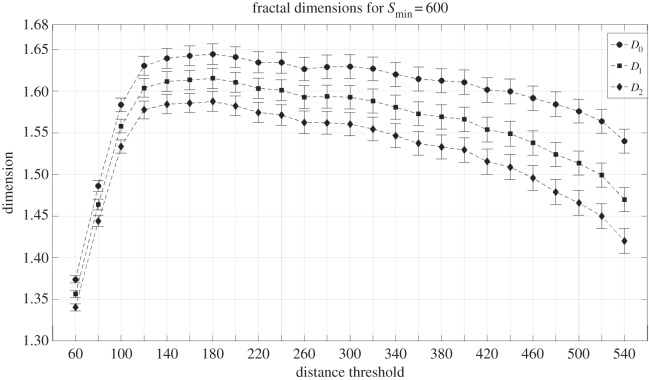
Fractal spectrum of clusters with a minimum size of $S_{\min}=600$ points obtained from the percolation on the intersection points.

**Figure 6.**
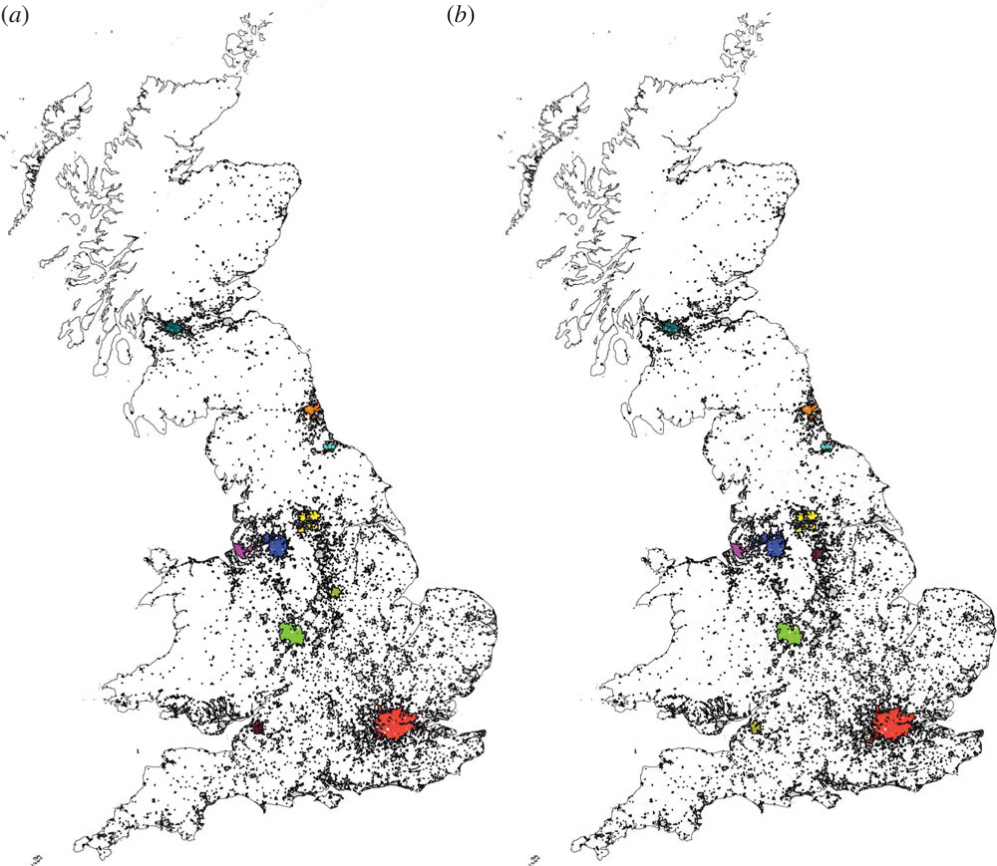
All clusters appearing at the maximum fractal value. (*a*) For the intersection points at *d*=180 m; (*b*) for the network at *d*=300 m, and black contours for the Corine dataset.

From the total spectrum, we select the three well-known fractal dimensions denoted by *D*_0_, *D*_1_ and *D*_2_; where *D*_0_ is the capacity dimension, and in practical terms it corresponds to the box-counting measure; *D*_1_ is the information dimension and it can be interpreted as Shannon’s entropy; and *D*_2_ is the correlation dimension, which is considered to be the most accurate one. A quick review of these measures can be found in the electronic supplementary material, appendix F. For the specific system at hand, we need to extract the characteristic fractal dimensions at each of the distance thresholds. Nevertheless, the clusters that emerge have all sorts of sizes, from individual points, to very large clusters. As we are interested in characterizing the urban space, and ultimately in recovering the cities of the system, which are the landmark of urbanization, we impose a minimum cluster size of 600 intersections for the computation of the fractal dimension. In addition, we do not compute the fractal properties beyond a maximum distance threshold of *d*=540 m, since the percolation method clearly returns regions beyond this transition, moving further and further away from a configuration of cities. The methodology to compute these three dimensions follows the same algorithms described in [53], where further details can be found. The results can be seen in figure 5. The evolution of all the three fractal properties of the system, indicates a maximum at *d*=180 m. Carefully inspecting the urban system at this maximum of its morphological characterization, we observe that the clusters at this distance threshold are in excellent correspondence with the classification of urbanized space given by the Corine dataset [51], which is a classification of the Landsat satellite images. The high level of agreement can be seen in figure 6, where the colour of the clusters are chosen according to size, and the black contours correspond to the classified urbanized areas.

#### 3.2.2 Clusters of networks

Let us now compute the fractal dimension of the clusters of networks that emerge from the percolation on the network. In this case, the fractal dimension *α* of the system is computed in terms of the scaling relationship between the mass of the clusters and the diameter of the network. The mass is given by the number of intersection points *N* and the diameter is denoted by $r_{\max}$, leading to

3.1 $$N∼r_{\max}^{\alpha }.$$

This corresponds to the same methodology implemented in [21]. Note that for this system we need to take a slightly larger maximum distance threshold *d*=800 m to ensure that we are well within the cities definition. In addition, in order to include as many small settlements as possible in the analysis, we use for networks a minimum cluster size of $S_{\min}=50$ nodes (a node is an intersection point), instead of 600.

**Figure 7.**
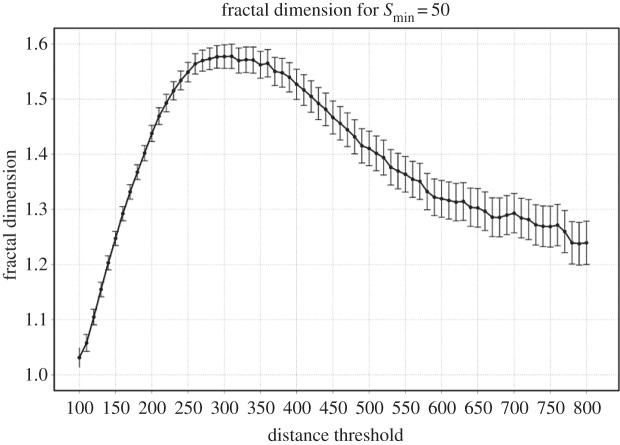
Fractal dimension of the whole urban system. It is computed on the networks obtained at different distance thresholds, using the scaling relationship between mass and diameter given by equation (3.1).

For this case, the results show a maximum at around *d*=300 m, see figure 7. Once again we see that the urban system defined at this maximum is in excellent correspondence with the definition of cities. Figure 6 shows the clusters at *d*=300 m, with the contours for the classified urbanized areas in black.

**Figure 8.**
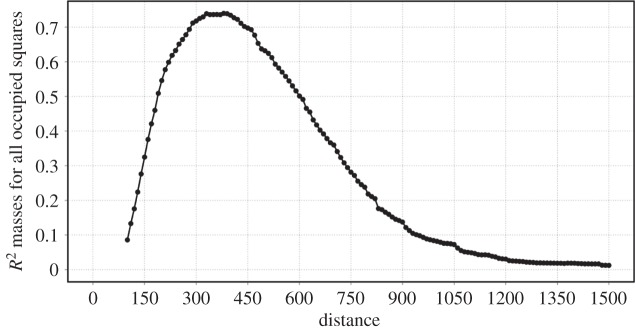
Correlation of the clusters from the network percolation with the boundaries of the Corine dataset.

We quantify the level of agreement between the clusters obtained through the percolation method and the urbanized areas in the Corine dataset through a correlation measure. Figure 8 indicates that the maximum of this correlation is also in the vicinity of *d*=300 m. Further details of how this measure is computed can be found in the electronic supplementary material, appendix D.

It is important to reiterate that the distance is not universal nor uniquely characterized. It is not universal, because it depends on the nature of the dataset. Hence a distance of *d*=300 m might suit this specific dataset for Britain, but might not suit another dataset, nor another European country. It is not uniquely defined, because the maximum corresponds to some sort of plateau. Hence any definition in the vicinity of *d*=300 m would be as accurate or as inaccurate as the one for *d*=300 m.

## 4. Conclusion

Throughout this work, we have shown that percolation theory can be applied to the street intersections and network, in order to uncover its intrinsic hierarchical structure. We argued that such an organization does not only relate to ideological or geographical divisions, but it represents a socio-economic polarization of the system. It has been extensively discussed that the regional development is tightly linked to the development of its infrastructure, hence it should not be surprising to find these economic patterns reflected in the density of the road network.

Regions are formed by settlements which share a stronger connectivity among themselves than among settlements of other regions. It is therefore not surprising to recover historical trajectories, and existing alliances. In this sense, looking at the highly integrated region of the cities in the north at *d*=540 m, it appears that the assimilation of Newcastle within the Northern Powerhouse proposal needs to be done with care, so that it is not left behind, given its weaker connectivity to the rest of the cities in the region. In this sense, many regional policies need to consider the strong ties that lie within the urban system.

From the perspective of the methodology, this formalism has the advantage that it can be implemented for incomplete datasets. We would have to test the robustness of the method with respect to the incompleteness of the dataset, but at this stage it is clear that the road intersections serve as a good proxy for urbanization [58], and that the percolation process on the point patterns recovers the hierarchical organization of the system. It is to be expected that the level of detail provided by the dataset would affect the level of detail for some of the transitions in the system, nevertheless, a hierarchical sketch can still be recovered. In addition, we have extrapolated the method to other spatial distributions where data are sparse, such as census data from the eleventh century, i.e. data from the Domesday Book.

Finally, this work has also provided a framework to define boundaries of cities in a global way, using a dataset that is open and not constrained to geographical units, such as the census data. In previous work, we developed a procedure to define cities using population density from the census [12]. Although successful, this procedure relies very heavily on data only available every 10 years, and on the level of granularity of the geographical unit. Nevertheless, it is still a useful method, since through the use of commuting data functional areas beyond urban cores can be defined, such as metropolitan areas. Note that a further refinement of the percolation approach can be found in [59], where each city is adjusted to its condensation threshold.

Future research is needed to understand the mechanisms that drive the system to a maximum fractal dimension at the point where the cities reach their urban extent.

## Supplementary Material

mapsSizeGrid_v1_0_20160212_2.eps

## Supplementary Material

Supplement Figures and appendix

## Supplementary Material

Reference

## Footnotes

1 This terminology adopted by the Chancellor of the Exchequer since June 2014 [42] has been incorporated into the vocabulary of politics, featuring in the main news agencies in the UK [43,44].

## Data accessibility

Data are available from Zenodo: http://dx.doi.org/10.5281/zenodo.46430.
