# Supplementary material for: Cities and regions in Britain through hierarchical percolation
Source: R Soc Open Sci. 2016 Apr 6;3(4):150691. doi: 10.1098/rsos.150691 (PMC4852634; doi:10.1098/rsos.150691)
Supplement: Supplement Figures and appendix [file rsos150691supp2.pdf]

Appendix

Algorithm for the percolation on the intersection points

The algorithm is based on the geographical location of intersections. We consider a pair of intersections as connected if they are no more than d meters apart. In order to reduce the computational complexity of the procedure, the actual analysis is performed using a grid of squared cells (10×10 meters each). A cell has one of two values: 1 if at least one intersection is within its area or *null* if it contains no intersections. As the percolation analysis is based on distance, we calculate a distance grid where each cell is assigned the distance to the closest cell that contains an intersection. We use this grid in the percolation procedure.

The percolation procedure for a distance d consists of the following steps:

(i) Each cell of the distance grid that has a distance value of d meters or below is marked as 1, otherwise, it is marked as *null*.
(ii) A unique identifier is assigned to each continuous set of marked cells. A cell is considered adjacent to its four nearest neighbours (i.e., its von Neumann neighbour).
(iii) Each intersection is assigned the unique identifier of its containing cell.

The method is implemented in ESRI ArcMap 10.1 using the following tools:

- The intersection grid is created using the *Points to Raster* tool.
- The distance grid is created using the *Euclidian distance* tool.
- The marked cells grid is created using the *Raster Calculator* tool.
- The unique identifiers grid is created using the *Region Group* tool.
- The unique identifiers are copied to the intersection points using the *Extract Values to Points* tool.

Algorithm for the network based percolation

Given a graph of the road network, where nodes represent intersections and the weight for each edge is the length of the street that connects them and a certain metric threshold (e.g. 5000m) we produce a network percolation via the following steps:

(i) We select the link of the graph with the smallest weight (distance), generating a new cluster and inserting both its nodes into the cluster.
(ii) We keep a first-in first-out queue of *nodes to expand*, from which we extract a node to continue the process. We add both nodes of the link selected in step 1 to this queue. Nodes are only added to this queue if they are not already included.
(iii) We extract a node from the queue of *nodes to explore* and if a link departing from that node (not yet included in the cluster) is smaller than the threshold, include the link in the cluster and the end node of the link in the queue of nodes to explore.
(iv) We repeat step 3 until no further node can be expanded (the queue is empty) and if there are links left in the graph that do not belong to any cluster, generate a new cluster by choosing the smallest available link and repeat from step 1.

Details of the clusters at the transitions

At each of the critical distances defining a transition, a set of clusters appears. There can be thousands of these, hence we opt to only visualise the 10 largest ones, setting colours representing a rank size: red is the biggest, blue is the second biggest, green the third etc. The rank and colour is illustrated in Fig. 4.

As discussed in the main text, although the critical distances differ for both systems, see Fig. 1, the intersection points and the network, the results are very similar. The maps for the transitions for the intersection points is given by Fig. S1, and and for the network by Fig. S2.

Correlation measure of the network percolation clusters and the urban area

For a given percolation result on the network, we categorise the type of each intersection as either being urban or non-urban. We define an urban intersection as an intersection that belongs to a cluster that is larger than Smin = 50 while the rest of the intersections are considered to be non-urban.

We use the polygons defined in the Corine dataset as a reference point. We generate a grid of 1Km per 1Km over the whole territory of the UK. For each square of the grid we assign two values, the first value is the area of the polygon that corresponds to the Corine cluster that intersects the square of the grid, the second value is the mass (the number of intersections) of the percolation cluster that has more intersections in the square of the grid. In order to be able to compare both systems we perform two types of analysis. The first is a Pearson Product Moment correlation between the values assigned to each square of the grid when there is both a cluster from the Corine and a percolation cluster. Fig. 8 shows that the highest correlation, for $R^2 > 0.7$ is given for the range of distances 300m to 400m. The second type is a measure of error comparing both. The procedure is as follows: when the squares of the grid do share both elements, we count the number of squares that have a Corine cluster but not a percolation cluster; and also the other way round, we count the number of squares that have a percolation cluster but not a Corine cluster. Finally we add both counts to get the total number of squares that do not have coincident clusters. The result is given in Fig. S3, and this shows that the total number of non-coincident clusters is minimised for $d = 300$m.

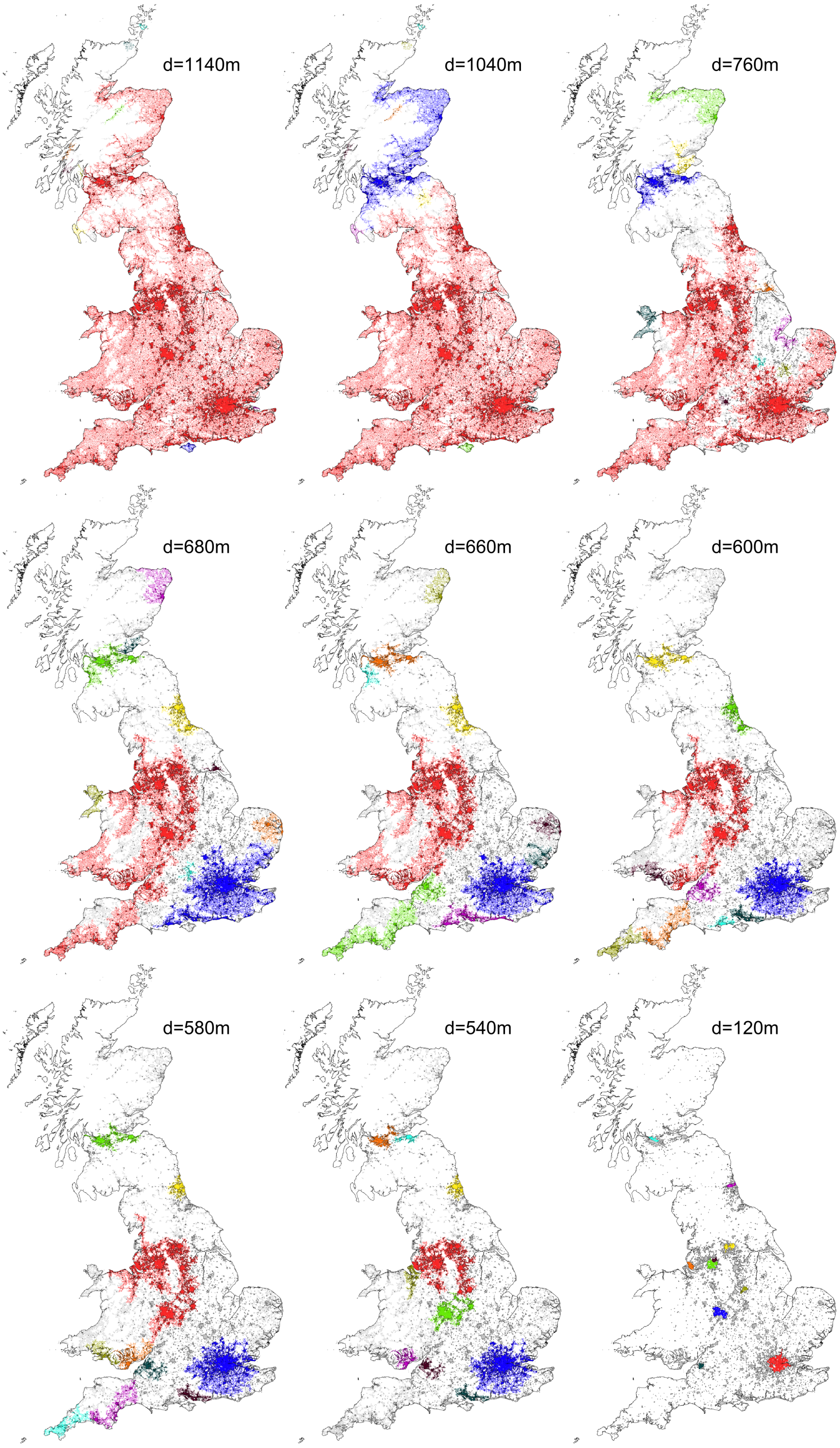

Figure S1. Maps of clusters at the transitions for the percolation on the intersection points. Only the 10 largest clusters have colours following the legend in the hierarchical tree.

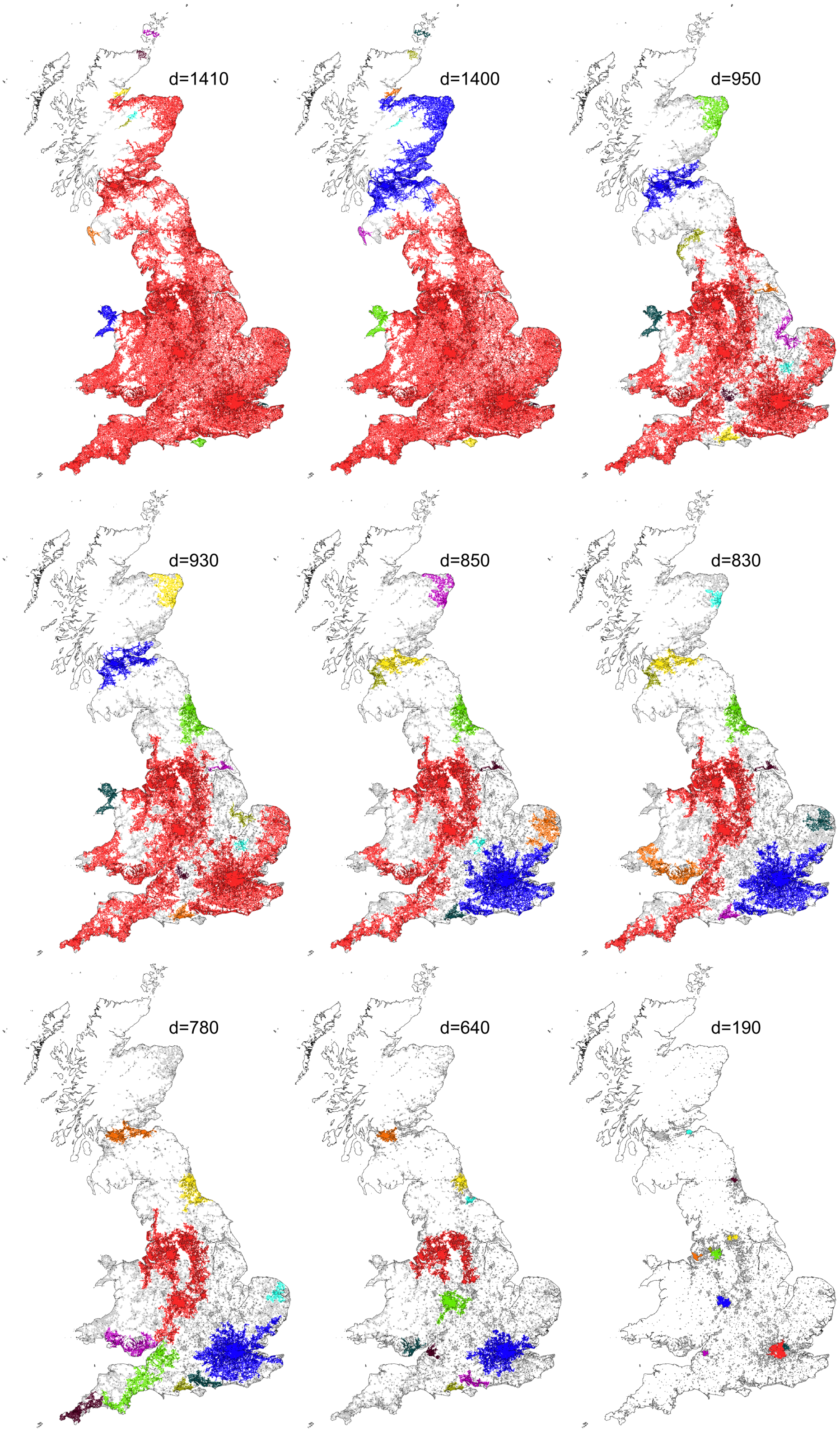

Figure S2. Maps of clusters at transitions for the network percolation. Only the 10 largest clusters have colours following the legend in the hierarchical tree.

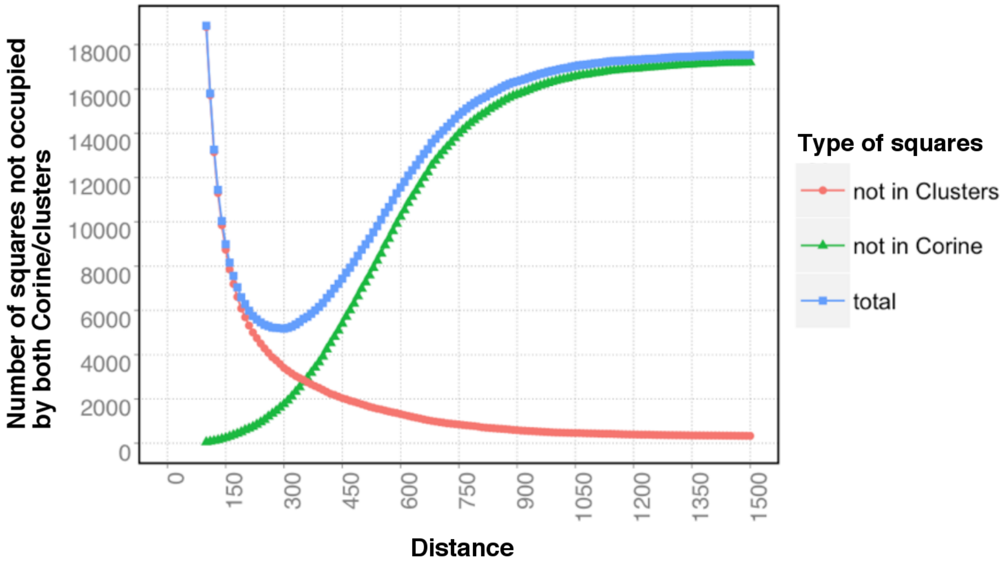

Figure S3. Measure of error of concurrency between the network percolation clusters and the urbanised clusters according to the Corine dataset.
