# Supplementary material for: Cities and regions in Britain through hierarchical percolation
Source: R Soc Open Sci. 2016 Apr 6;3(4):150691. doi: 10.1098/rsos.150691 (PMC4852634; doi:10.1098/rsos.150691)
Supplement: Reference [file rsos150691supp3.docx]

**References**

60. Clauset A, Shalizi CR, Newman MEJ. 2009 Power-law distributions in empirical data. *SIAM. Rev*. **51**, 661-703. (doi:[10.1137/070710111](http://dx.doi.org/doi:10.1137/070710111))
